# Impaired Inactivation of L-Type Ca^2+^ Current as a Potential Mechanism for Variable Arrhythmogenic Liability of HERG K^+^ Channel Blocking Drugs

**DOI:** 10.1371/journal.pone.0149198

**Published:** 2016-03-01

**Authors:** Jae Gon Kim, Dong Jun Sung, Hyun-ji Kim, Sang Woong Park, Kyung Jong Won, Bokyung Kim, Ho Chul Shin, Ki-Suk Kim, Chae Hun Leem, Yin Hua Zhang, Hana Cho, Young Min Bae

**Affiliations:** 1 Department of Physiology and the Samsung Biomedical Research Institute, Sungkyunkwan University School of Medicine, Suwon, South Korea; 2 Next-Generation Pharmaceutical Research Center, Korea Institute of Toxicology, Korea Research Institute of Chemical Technology, Daejeon, South Korea; 3 Human and Environmental Toxicology Program, University of Science and Technology, Daejeon, South Korea; 4 Division of Sport Science, College of Science and Technology, Konkuk University, Choongju, South Korea; 5 Department of Physiology, KU Open Innovation Center, Research Institute of Medical Science, Konkuk University School of Medicine, Chungju, South Korea; 6 Department of Veterinary Pharmacology and Toxicology, College of Veterinary Medicine, Konkuk University, Seoul, South Korea; 7 Department of Physiology, University of Ulsan College of Medicine, Seoul, South Korea; 8 Department of Physiology, Seoul National University College of Medicine, Seoul, South Korea; National University of Singapore, SINGAPORE

## Abstract

The proarrhythmic effects of new drugs have been assessed by measuring rapidly activating delayed-rectifier K^+^ current (*I*_Kr_) antagonist potency. However, recent data suggest that even drugs thought to be highly specific *I*_Kr_ blockers can be arrhythmogenic via a separate, time-dependent pathway such as late Na^+^ current augmentation. Here, we report a mechanism for a quinolone antibiotic, sparfloxacin-induced action potential duration (APD) prolongation that involves increase in late L-type Ca^2+^ current (*I*_CaL_) caused by a decrease in Ca^2+^-dependent inactivation (CDI). Acute exposure to sparfloxacin, an *I*_Kr_ blocker with prolongation of QT interval and torsades de pointes (TdP) produced a significant APD prolongation in rat ventricular myocytes, which lack *I*_Kr_ due to E4031 pretreatment. Sparfloxacin reduced peak *I*_CaL_ but increased late *I*_CaL_ by slowing its inactivation. In contrast, ketoconazole, an *I*_Kr_ blocker without prolongation of QT interval and TdP produced reduction of both peak and late *I*_CaL_, suggesting the role of increased late *I*_CaL_ in arrhythmogenic effect. Further analysis showed that sparfloxacin reduced CDI. Consistently, replacement of extracellular Ca^2+^ with Ba^2+^ abolished the sparfloxacin effects on *I*_CaL_. In addition, sparfloxacin modulated *I*_CaL_ in a use-dependent manner. Cardiomyocytes from adult mouse, which is lack of native *I*_Kr_, demonstrated similar increase in late *I*_CaL_ and afterdepolarizations. The present findings show that sparfloxacin can prolong APD by augmenting late *I*_CaL_. Thus, drugs that cause delayed *I*_CaL_ inactivation and *I*_Kr_ blockage may have more adverse effects than those that selectively block *I*_Kr_. This mechanism may explain the reason for discrepancies between clinically reported proarrhythmic effects and *I*_Kr_ antagonist potencies.

## Introduction

Drug-induced QT interval prolongation and the appearance of torsade de pointes (TdPs) are recognized as potential risks associated with the use of a wide range of noncardiovascular drugs including antibiotics [1–4]. Quinolone antibiotics have been suggested to have a class effect of blocking the human *Ether-a-go-go-*related gene (hERG) K^+^ channel expressing the rapid component of the delayed rectifier current (*I*_Kr_) in the human heart, and thus prolong action potential duration (APD), which is associated with QT interval prolongation. The quinolone antibiotic sparfloxacin (SPX) has been withdrawn from U.S. drug market, because it was shown to induce QT interval prolongation and ventricular arrhythmia [5, 6]. Another quinolone, grepafloxacin, was withdrawn because it induced TdP, a polymorphic ventricular tachycardia (VT) linked to excessive QT interval prolongation [7]. Concern over the proarrhythmic effects of many other quinolone antibiotics continues to grow. In nonclinical studies, the proarrhythmic effects of clinically used quinolone antibiotics have been assessed by measuring their associated *I*_Kr_ antagonist potency. However, discrepancies between clinically reported proarrhythmic effects and in vitro observations exist. For example, the antibiotic moxifloxacin blocks *I*_Kr_ but has been associated with drug-induced long QT syndrome (LQT) only very rarely [8, 9].

Cardiac rhythm and contractility are regulated by the composite functions of cardiac myocyte ion channels. Specifically, the lengthening and flattening of action potential (AP) plateaus are determined by the sum of inward and outward currents. *I*_CaL_ contribute to inward currents, maintaining the plateau phase of ventricular AP (phase 2). Inhibition of *I*_CaL_ shortens the AP, whereas inhibition of outward *I*_Kr_ results in AP prolongation. Therefore, if both K^+^ and Ca^2+^ channels are inhibited, *I*_CaL_ inhibition may counteract the *I*_Kr_-blocking effects of quinolone antibiotics. Indeed, Xu *et al*.(2003) reported that drugs with dual blocking action against hERG K^+^ and Ca^2+^ channels are less likely to cause arrhythmias than drugs with selective blocking activity against hERG K^+^ current [10]. On the other hand, enhancing *I*_CaL_ while blocking *I*_Kr_ may aggravate APD prolongation and/or generate early afterdepolarization (EAD) upstrokes.

During a normal AP, *I*_CaL_ peaks early, triggering robust sarcoplasmic reticulum Ca^2+^ release before partially inactivating due to two processes: Ca^2+^-dependent inactivation (CDI), mediated by Ca^2+^ binding to calmodulin (CaM) tethered to the C-terminus of the channel, and voltage-dependent inactivation (VDI). The rate and degree of *I*_CaL_ inactivation due to these processes during the late phase of the APD has a major effect on repolarization. This raises the possibility that instead of potentiating *I*_CaL_, modifying its shape by altering its inactivation kinetics might lead to APD prolongation and EAD. In a recent study, elimination of CDI in guinea pig ventricular myocytes via expression of Ca^2+^-insensitive CaM (CaM1234) was shown to produce “ultralong” Aps [11].

In the present study, we sought to determine the relevance of *I*_CaL_ in APD prolongation effects of SPX. We analyzed the biophysical properties of L-type Ca^2+^ channels affecting APD. SPX reduced peak *I*_CaL_. However this quinolone antibiotic augmented late *I*_CaL_ by attenuating CDI, which promoted APD prolongation in cardiac myocytes. In contrast, *I*_Kr_ blockers not associated with serious arrhythmias—ketoconazole, ciprofloxacin, enoxacin, ofloxacin, and levofloxacin produced no change in *I*_CaL_ or decreased both peak and late *I*_CaL_, suggesting the role of increased late *I*_CaL_ in arrhythmogenic effect. Our results suggest an importance of calcium channel inactivation in producing the arrhythmogenic effects of SPX and, as such, it is necessary to consider *I*_CaL_ property changes when assessing drugs for QT prolonging and arrhythmogenic liability.

## Materials and Methods

### Cardiomyocyte isolation and culture

All animal care and experimental procedures complied with the National Institutes of Health guidelines, and the Institutional Animal Care and Use Committee of Konkuk University and Sungkyunkwan University approved this study. Neonatal ventricular myocytes were isolated from 1 to 2-day-old Sprague-Dawley (SD) rats (Nara Biotech, Seoul, Korea) by using a previously reported method [12]. Ventricular regions of neonatal rat hearts were excised (approximately, the lower third) and the tissues (approximately 1–2 mm) were minced on ice. The minced tissues were treated with a solution containing 0.1% collagenase (Wako, Japan), 0.1% trypsin, and 1% glucose in phosphate-buffered saline (Ca^2+^/Mg^2+^-free) at 37°C for 10 min. After the supernatant from the first digestion was removed, three 10-min digestions were performed using the same enzyme solution. The supernatants were stored in DMEM/F-12 culture medium containing 10% fetal bovine serum, 5% horse serum, penicillin-streptomycin (100 U/ml and 100 μg/ml, respectively) in a 4°C ice chamber and the centrifuged for 7 min at 700 × *g*. The cell pellets were incubated at 37°C in a 95% O_2_ incubator for 1.5 h to attach non-cardiac myocytes to microscope cover glass. The cells were subsequently cultured on the cover glass for 3 days at 37°C and 95% O_2_. Cells cultured for 3–5 days were used for *I*_CaL_ current recordings.

Ventricular myocytes from adult mice (> 5 months) or 2-week-old SD rats were isolated as previously described with minor modifications [13]. Briefly, ventricular myocytes were isolated by perfusing Ca^2+^-free NT solution containing collagenase (1 mg/ml, 4176, Worthington) and DL-dithiothreitol (1 mg/ml, D-0632, Sigma, St. Louis, MO, USA) through Langendorff columns at 37°C.

### Electrophysiological recording

Cardiac myocytes were subjected to patch-clamp experiments. Whole-cell Ca^2+^ and Ba^2+^ currents were recorded using a conventional whole-cell patch-clamp configuration, outfitted with an EPC 8 patch-clamp amplifier (Heka, Germany). Voltage pulse generation was controlled using R-clamp software (R-clamp; provided by Dr. S.Y. Ryu). The data were digitized using the R-clamp software at a sampling rate of 5 kHz, after being low-pass filtered at 1 kHz. The patch pipettes were created from borosilicate glass capillaries (Clark Electromedical Instruments, Pangbourne, UK) by using a puller (PP-83; Narishige, Japan). Patch pipettes producing resistances of 1.5–2.5 MΩ in bathing solution were used. All experiments were performed at room temperature (20–25°C).

APs were recorded using a nystatin-perforated patch-clamp configuration, with an EPC10 patch-clamp amplifier (Heka, Germany). Data were digitized and current injection (125–175 pA, 9 ms) for AP generation were both controlled using Patch-Master software.

### Solutions and Drugs

To record *I*_CaL_ in cardiac myocytes, NMDG-Tyrode’s [143 mM *N*-methyl-d-glucamine (NMDG)-Cl, 5.4 mM CsCl, 0.33 mM NaH_2_PO_4_, 5 mM HEPES, 0.5 mM MgCl_2_, 1.8 mM CaCl_2_, 11 mM d-glucose, pH adjusted to 7.4 with HCl] was used as the bathing solution. The pipette solution contained 115 mM CsCl, 5 mM Mg-ATP, 10 mM HEPES, 5 mM ethylene glycol-bis (2-aminoethylether)-*N*,*N*,*N*,*N*,-tetraacetic acid (EGTA), and 5 mM creatine phosphate (disodium salt). The pH was adjusted to 7.3 by using CsOH. APs were recorded from single isolated myocytes in a perforated patch configuration by using nystatin (200 μg/ml). Normal Tyrode’s solution (143 mM NaCl, 5.4 mM KCl, 0.33 mM NaH_2_PO_4_, 5 mM HEPES, 0.5 mM MgCl_2_, 1.8 mM CaCl_2_, 11 mM d-glucose, pH adjusted to 7.4 with NaOH) was used as the bathing solution. The pipette solution for recoding APs contained 140 mM KCl, 10 mM HEPES, 5 mM EGTA, and 1 mM MgCl_2_, and the pH was adjusted to 7.2 with KOH.

To record I_Na_ in cardiac myocytes, 140 mM NaCl, 5 mM CsCl, 1.8 mM CaCl_2_, 1 mM MgCl_2_, 11 mM Glucose, 10 mM HEPES adjusted with NaOH (pH 7.4) was used as the bath solution. Nifedipine (1 μM), SN-6 (10 μM), and CdCl_2_ (100 μM) were used to block L-type Ca^2+^ currents, NCX currents, and T-type Ca^2+^ currents, respectively. The pipette solution contained 20 mM CsCl, 100 mM Cs-Asp, 10 mM EGTA, 10 mM HEPES, 20 mM TEA-Cl, 5 mM Mg-ATP adjusted to 7.25 with CsOH.

Unless otherwise stated, all chemicals and drugs were purchased from Sigma-Aldrich; SPX (Fluca, 56968) and E4031 (Sigma, M5060) were prepared as stock solutions in dimethyl sulfoxide. The drugs were diluted in the bathing solution on the day of the experiment.

### Statistical analysis

The results are shown as mean ± standard error of the mean. Student’s *t*-tests or Fisher's exact test were performed to test for significance as appropriate using SigmaPlot. P values <0.05 were deemed to be statistically significant.

## Results

### SPX induces APD prolongation in the presence of *I*_*Kr*_ blocker

**Fig 1:**
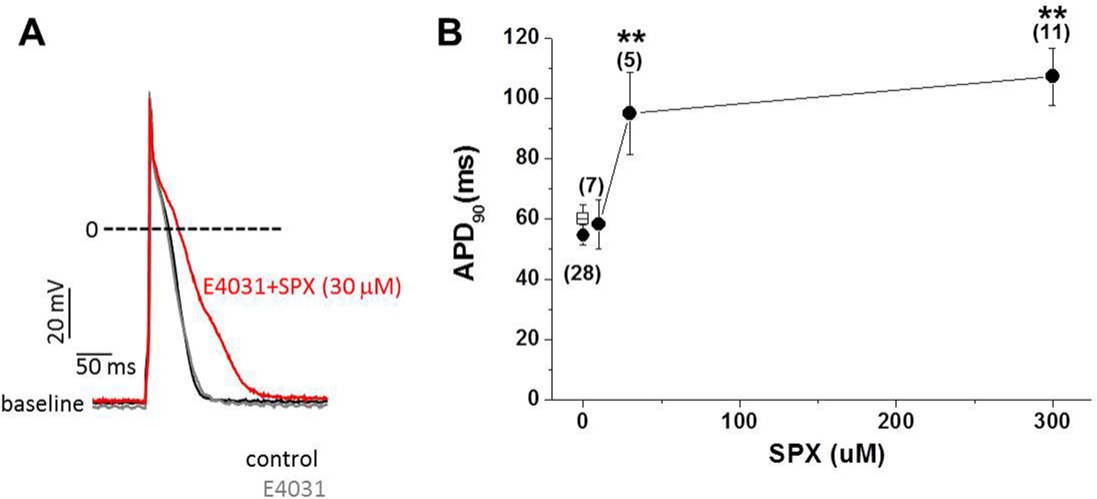
The effect of SPX on APs recorded in the presence of E4031. Action potentials were recorded by pacing myocytes at 2 Hz. In the presence of 10 μM E4031, SPX (10–300 μM) was applied. Data were obtained at 37°C by using the perforated patch technique. A, Examples of action potentials recorded before (gray) and after (red) SPX exposure at a concentration of 30 μM. The control action potential (black) before E-4031 treatment was also overlaid for comparison. B, APD_90_ was plotted as a function of SPX concentration. Asterisks indicate statistical significance (paired *t*-test; *P < 0.05, **P < 0.01). Error bars indicate standard error. The numbers in parentheses indicate the number of cells tested.

To test the hypothesis that factors other than *I*_Kr_ modulation play important roles in SPX- induced APD prolongation, the effects of SPX applied at concentrations between 10 and 300 μM were investigated in 2-week-old rat ventricular myocytes in the presence of E4031, a selective *I*_Kr_ blocker. APs were elicited by electrical stimulations delivered using a patch pipette in current clamp mode, at a stimulation frequency of 2 Hz. As shown in Fig 1A, 30 μM SPX significantly prolonged APD in the presence of 1 μM E4031. The time required for 90% repolarization (APD_90_), increased from 54.5 ± 3.3 to 95.0 ± 13.6 ms after 10 min of 30-μM SPX treatment (paired *t*-test; n = 7, P < 0.05).

No changes in resting membrane potential (RMP) or in AP overshoot potentials were observed during SPX treatments. The mean RMP and overshoot potential values after SPX treatment were −68.5 mV (n = 7; P > 0.05 vs. −70.8 mV in the absence of SPX) and 53.5 mV (n = 7; P > 0.05 vs. 52.7 mV in the absence of SPX), respectively. The steady-state APD_90_ values obtained at various SPX concentrations are summarized in Fig 1B. These data indicate that SPX-induced APD prolongation is not only attributable to blocking *I*_Kr_, but is also influenced by additional channel modulation.

### Effects of SPX on *I*_CaL_

**Fig 2:**
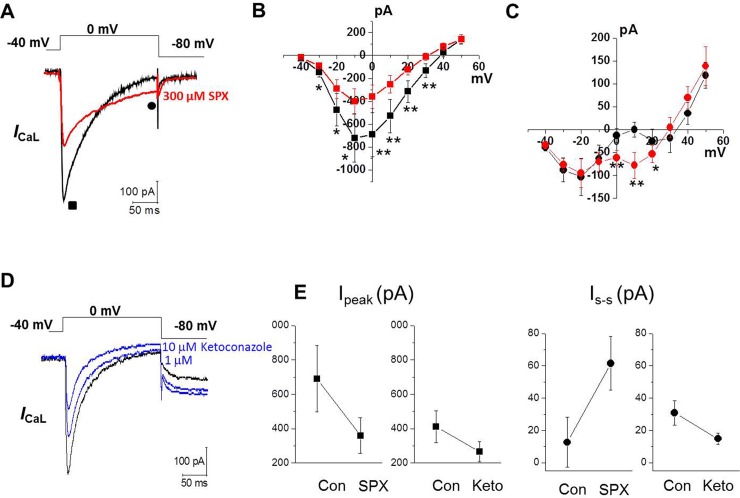
Effect of SPX on *I*_CaL_. A, Representative traces of *I*_CaL_ during a 200-ms voltage-clamp pulse from −40 to 0 mV before (black) and after (red) exposure to 300 μM SPX. B–C, current–voltage (*I*-V) relationships of *I*_peak_ (■, B) and *I*_end of pulse_ (●, C) under control conditions (black) and after application of 300 μM SPX (red) (n = 10). D, Representative traces of *I*_CaL_ to 1 μM and 10 μM of ketoconazole (blue) (n = 4). E, The comparison of *I*_peak_ (■, left) and *I*_s-s_ (●, right) from *I*_CaL_ before and after 300 μM SPX (n = 10) or 10 μM ketoconazole (n = 4). *P < 0.05 **P < 0.01.

We next examined whether SPX enhances *I*_CaL_ in neonatal cardiomyocytes. By holding E_m_ at −50 mV, we could successfully isolate *I*_CaL_ from *I*_CaT_ measurements (S1 Fig). Therefore, we used holding potential of −50 mV for all voltage-clamp experiments, excluding those reported in Fig 2, in which we applied a double pulse at −40 mV followed by 0 mV from a holding potential of −80 mV [14]. To eliminate voltage-gated K^+^ currents and Na^+^ currents, a Cs^+^-rich pipette solution and Na^+^-free (substituted with NMDG^+^) bath solution was used. Fig 2A shows representative *I*_CaL_ measurements under control conditions and in the presence of 300 μM SPX, recorded from the same ventricular myocytes. The amplitude of the peak *I*_CaL_ was reduced after SPX treatment. The *I*–V curves indicated that the peak *I*_CaL_ was decreased after 300 μM SPX treatment at all potentials ranging from −40 to +50 mV, without altering the *I*–V relationship (n = 10; Fig 2B). Inactivation, however, was slowed by SPX treatment. Slower *I*_CaL_ decay values resulted in larger current amplitudes in the presence of SPX at the end of the 200 ms pulse (Fig 2C). The amplitude of this current was larger over the voltage range 0 to +20 mV—i.e. the range in which the AP plateau typically occurs.

We also examined the effects on *I*_CaL_ of multiple *I*_Kr_ blockers that have not been associated with severe arrhythmias. Ciprofloxacin, enoxacin, ofloxacin, and levofloxacin are quinolone antibiotics with variable *I*_Kr_ potencies that are rarely associated with LQTS risk [15, 16]. They had no effect on *I*_CaL_ (S2 Fig). We also examined the effect on *I*_CaL_ of ketoconazole an antifungal agent which is known to block *I*_Kr_ but is not associated with TdP risk [17, 18]. Ketoconazole did decrease *I*_CaL_ (Fig 2D). In contrast to SPX, however, it reduced late *I*_CaL_ as well as peak *I*_CaL_ (Fig 2E). Taken together, these data suggest that SPX increased late *I*_CaL_ that might be related with its ability to induce arrhythmias.

### Effect of SPX on the Inactivation Kinetics of *I*_*CaL*_

**Fig 3:**
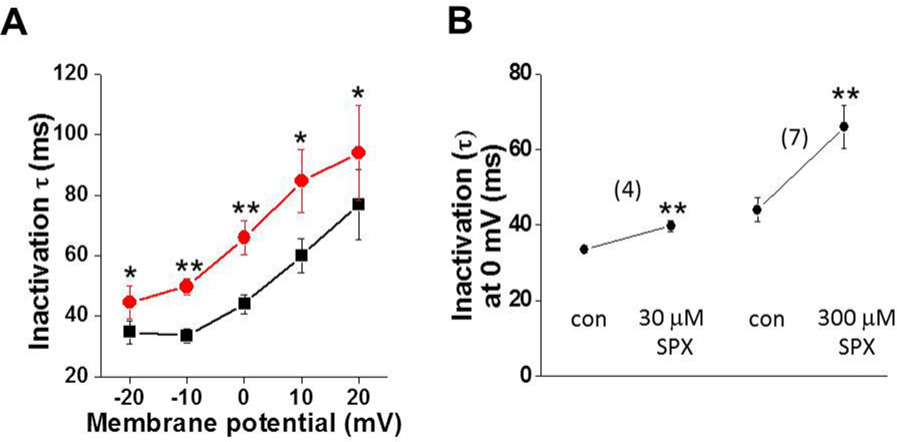
Effects of SPX on I_CaL_ inactivation time course. A, Summary of the effects of SPX (300 μM) on the time course (time constants, τ) of inactivation at various membrane potentials (n = 4–5). B, Summary of the concentration-dependent slowing of *I*_CaL_ inactivation by SPX at 0 mV (n = 4–5). *P < 0.05 **P < 0.01.

To further investigate the effect of SPX on inactivation kinetics, the decay phase of *I*_CaL_ was fitted using an exponential function. When the decay phase was fitted using a monoexponential function, the time constant (τ) was significantly increased by SPX treatment (Fig 3A). The voltage dependence of τ remained unchanged in the presence of SPX. As shown in Fig 3B, the effects of SPX on inactivation kinetics were dose-dependent. Taken together, these results suggest that SPX induces APD prolongation by slowing *I*_CaL_ inactivation.

### SPX reduces Ca^2+^-dependent *I*_CaL_ inactivation

**Fig 4:**
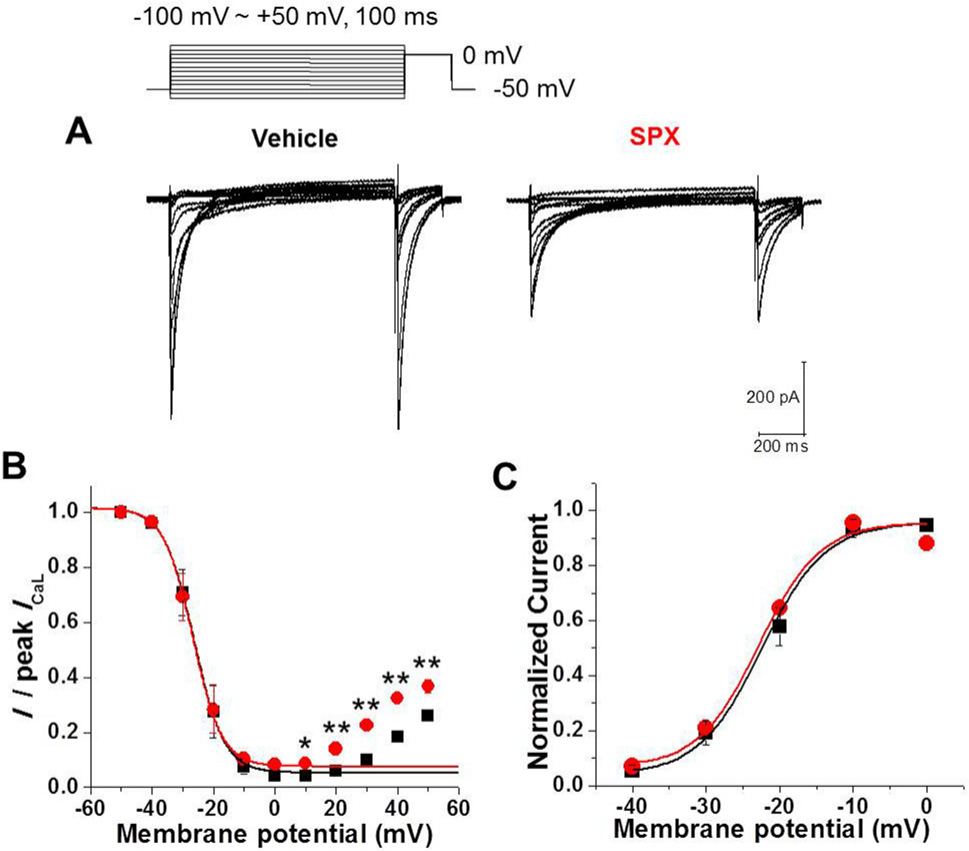
Effect of SPX on the steady-state inactivation of *I*_CaL_. A, The steady-state inactivation levels were measured by using double-pulse protocol. B, Steady-state inactivation curves for the *I*_CaL_ in the absence and presence of SPX (300 μM). C, Voltage dependence of *I*_CaL_ activation in the absence and presence of SPX (300 μM). The half-activation voltage was not significantly changed by SPX (n = 10, paired *t*-test, P > 0.05).

The effects of SPX on late *I*_CaL_ inactivation were investigated using conventional double-pulse protocol. Prepulses 1000 ms in duration and at various potentials ranging from −50 to +50 mV in 10 mV steps preceded a 100-ms test pulse at 0 mV. The superimposed current responses to the test pulse (0 mV) are shown in Fig 4A (left, control; right, 300 μM SPX). The slow inactivating *I*_CaL_ in the presence of SPX indicates that SPX exerts a pharmacological effect (Fig 4A, right).

Under control conditions, inactivation increased sharply as the prepotential increased from −40 to −20 mV, reaching a maximum of ~95.8% at +10 mV (Fig 4B). At prepulse potentials greater than +20 mV, the extent of inactivation decreased, resulting in a U-shaped *I*_Ca,L_ inactivation curve. The data from −100 to +10 mV were fitted using the following Boltzmann equation:

$$\text{y}=(A_{1}\;-A_{2})\;/\left\{1+\text{exp}\;\left[\frac{\left(V-V_{1/2}\right)}{\kappa }\right]\right\}+A_{2}$$

where *V* is the membrane potential, *V*_1/2_ is the membrane potential of half-maximum inactivation, and *k* is the slope of the inactivation curve. *A*_1_ represents the maximal amplitude and *A*_2_ is the amplitude of the non-inactivating component of *I*_Ca,L_. *V*_1/2_ was −26.2 ± 1.4 mV and *k* was +4.9 ± 0.7 mV under control condition.

The current availability curves produced in the presence of SPX (Fig 4B) indicate that SPX significantly reduced steady-state inactivation. When the data from −100 to +10 mV were fitted using the Boltzmann equation, the *A*_2_ value, the amplitude of the non-inactivating component of *I*_Ca,L_, was increased by SPX (0.087 ± 0.008 vs. 0.042 ± 0.012 in control, n = 5, P < 0.01). Neither *V*_1/2_ nor *k* were affected (-26.6 ± 1.5 mV and +4.8 ± 0.8 mV, respectively; P > 0.05). In addition, at prepulse potentials greater than 0 mV, *I*_CaL_ amplitudes in the presence of SPX were greater than those under control conditions (P < 0.01; paired *t*-test; n = 5).

We confirmed that no differences in the steady-state activation curves (Fig 4C) were present before and after SPX treatment. The voltages for half-activation were −22.2 ± 0.1 mV (n = 10) in the control and −23.2 ± 1.2 mV (n = 10) in the presence of SPX (paired *t*-test, P > 0.05). These data suggest that SPX attenuates inactivation, leading to slower *I*_CaL_ decay.

**Fig 5:**
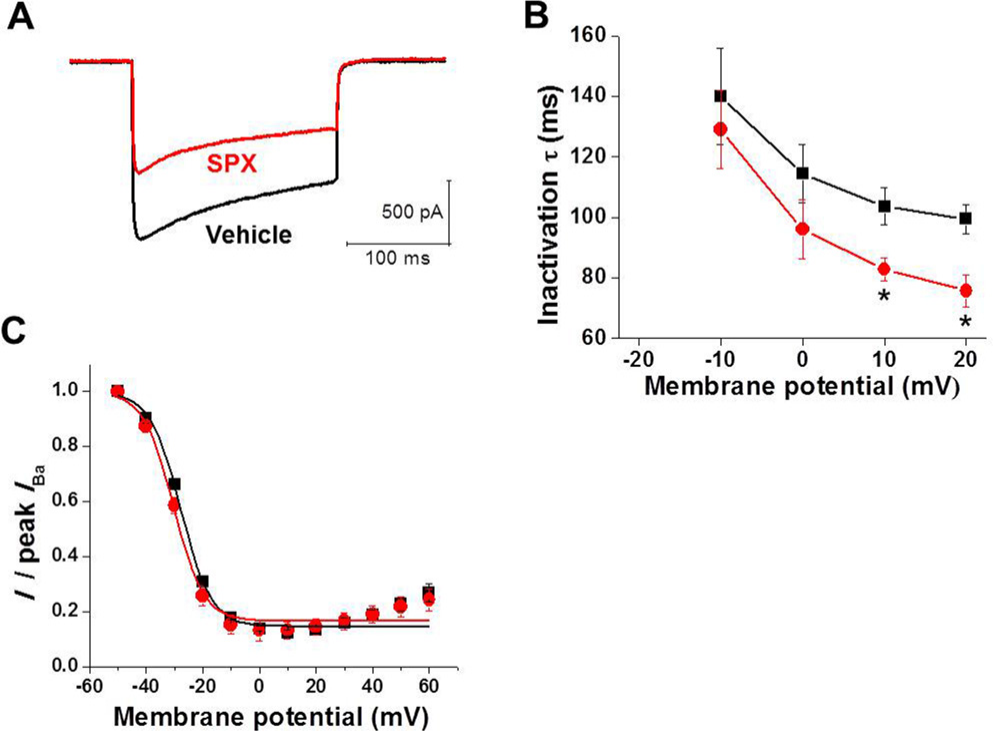
Effects of SPX on Ba^2+^ currents. A, Representative traces of *I*_CaL_ with external Ba^2+^ replacing Ca^2+^ as charge carrier during a 200 ms voltage-clamp pulse from −50 to 0 mV before (black) and after (red) exposure to 300 μM SPX. B, Voltage-dependence of Ba^2+^ current inactivation time constants (τ) under control conditions (black) and in the presence of 300 μM SPX (red). Data points (mean ± standard error) are from 5 cells. C, The steady-state inactivation curves of Ba^2+^ currents under control conditions (black) and in the presence of 300 μM SPX (red) (n = 3). *P < 0.05.

L-type Ca^2+^ channels can be inactivated by two different mechanisms: CDI and VDI. The Ca^2+^-dependent aspect of L-type Ca^2+^ channel inactivation is dependent on Ca^2+^ entry. Therefore, essentially all inactivation of Ba^2+^ current through the L-type Ca^2+^ channels is voltage dependent. The substitution of Ba^2+^ ions for Ca^2+^ has been used widely to separate the contribution VDI from CDI to the macroscopic *I*_CaL_. We confirmed the results reported previously, showing the Ca^2+^ dependence of CDI in our cells and that the effects of SPX are changed by replacing Ca^2+^ with Ba^2+^ (Fig 5). As shown in Fig 5A, SPX did not slow Ba^2+^ current inactivation. SPX did not increase the inactivation time constant, but instead reduced it, indicating that the attenuation of *I*_CaL_ inactivation by SPX was abolished (Fig 5B). In addition, SPX had little effect on the steady-state inactivation of Ba^2+^ currents (Fig 5C). These results confirm that SPX specifically modulates CDI.

### Use-dependency

**Fig 6:**
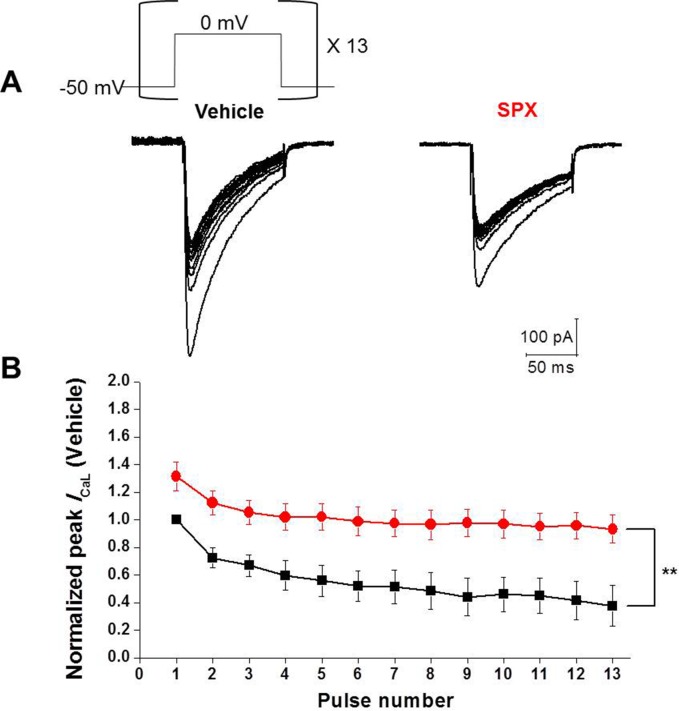
Use-dependency of SPX on *I*_CaL_. A, Repetitive application of depolarizing pulses (shape is shown in the figure inset) to 0 mV from a holding potential of −50 mV gradually decreased the *I*_CaL_ amplitude. The frequency of depolarizing pulses was 2 Hz. In the presence of SPX, the gradual decrease of *I*_CaL_ was significantly attenuated. B, Summary of the use-dependent effect of SPX. Normalized current levels are plotted against pulse number (A and B, n = 6; black, control; red, SPX). The amplitudes of steady-state currents of both control and SPX group were normalized by the first current of the control. *P < 0.05 **P < 0.01 (n = 6).

Since SPX slowed the inactivation of *I*_CaL_ by inhibiting CDI, it is expected that repetitive application of depolarizing voltage steps may cause less accumulation of *I*_CaL_ inactivation in the presence of SPX. In order to prove this hypothesis, a series of depolarizing step pulses at frequencies of 2Hz were applied (Fig 6A, inset). Fig 6A and 6B shows superimposed current traces of cells in the absence and presence of SPX, respectively. When the depolarizing step pulses were applied repetitively under control conditions, the *I*_CaL_ peak amplitudes were gradually decreased (50.6 ± 7.3% of the initial level at pulse 13; Fig 6C). In the presence of SPX (300 μM), however, this gradual decrease was significantly attenuated, with the *I*_CaL_ peak amplitude being 63.3 ± 5.7% of the initial level by pulse 13 (Fig 6C). Fig 6C illustrates the gradual decrease of *I*_CaL_ peak amplitudes during repetitive pulses in the absence and presence of SPX.

### Recovery from inactivation

**Fig 7:**
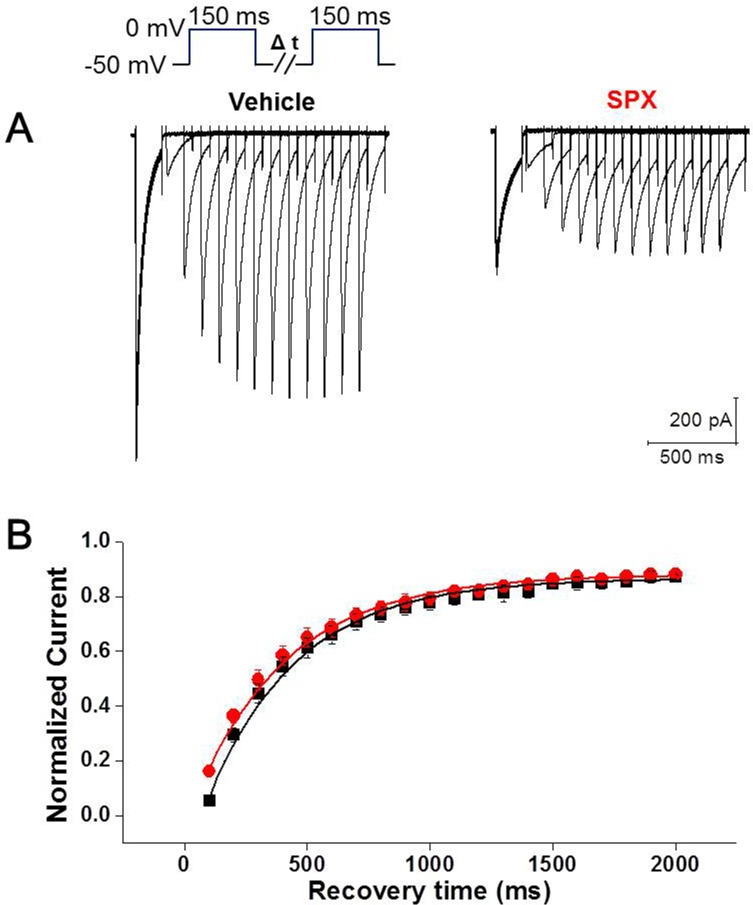
Effect of SPX on time course of the recovery from inactivation. A, Raw current tracings in the absence and presence of SPX. The voltage pulse protocol is shown as a figure inset. B, Summary of time courses of the recovery from inactivation in the absence and presence of SPX (n = 6; black, vehicle; red, SPX). *P < 0.05 **P < 0.01.

Inactivation and recovery from inactivation are closely related processes and are critical factors that determine channel function. Recovery from inactivation was investigated by eliciting sustained depolarization (200 ms), followed by recovery intervals of increasing durations, and then applying a subsequent test pulse (Fig 7A, inset). In comparison to the control, recovery from inactivation was not changed by SPX treatment (Fig 7A and 7B). Data were fitted to a single exponential function. The time constants for recovery from inactivation were 365.4 ± 20.0 ms (n = 7) in the control and 382.6 ± 22.9 ms (n = 7) in the presence of SPX (paired *t*-test, P > 0.05).

### Effect of SPX on *I*_*CaL*_ and AP in adult mouse cardiac myocytes

**Fig 8:**
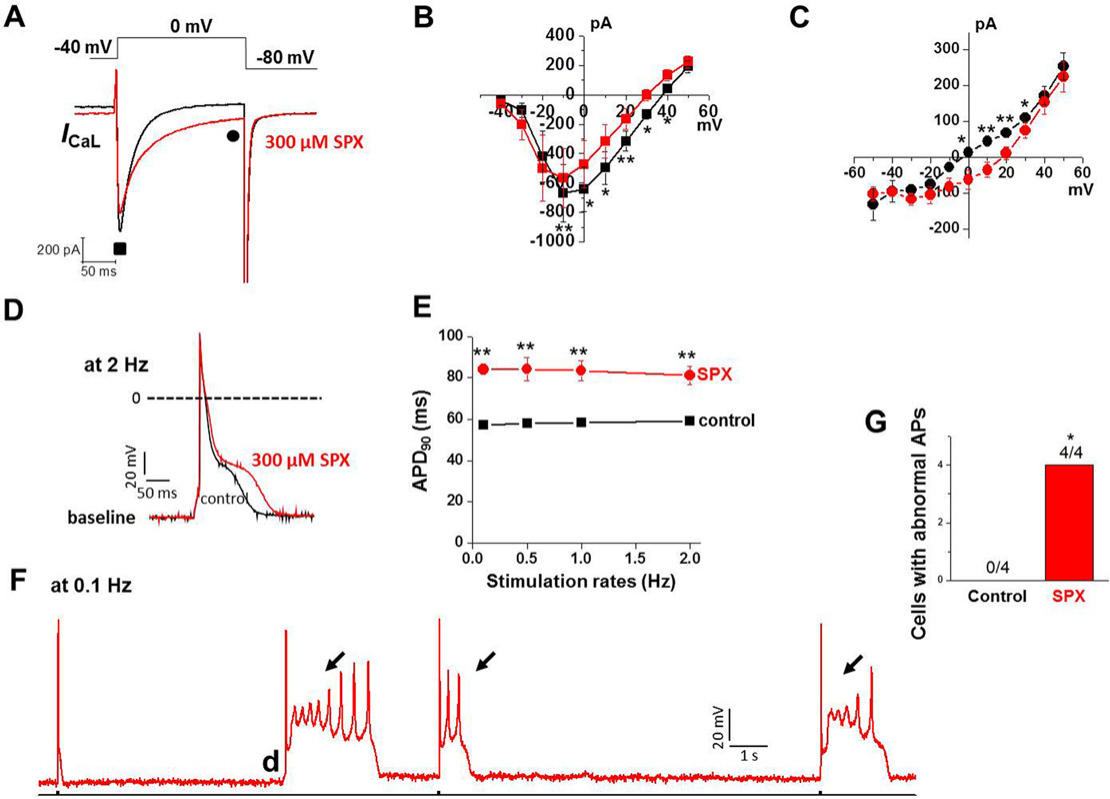
Effect of SPX on *I*_CaL_ and AP in adult mouse cardiac myocytes. A, Representative traces of *I*_CaL_ during a 200-ms voltage-clamp pulse from −40 to 0 mV before (black) and after (red) exposure to 300 μM SPX. B–C, current–voltage (*I*-V) relationships of *I*_peak_ (■, B) and *I*_end of pulse_ (●, C) under control conditions (black) and after application of 300 μM SPX (red) (n = 4). D. SPX prolonged APs of adult mouse cardiomyocyte at a stimulation rate of 2 Hz. E, Plot of APD_90_ over a range of stimulation rate in adult mouse cardiomyocytes in the absence and presence of SPX. F. Example of AP recorded after 30 min exposure to SPX at a slow stimulation rate (0.1 Hz). EADs (arrows) and delayed afterdepolarization (DAD; d) were observed, G. Summary data showing frequency of afterdepolarizations in control and SPX-treated cells. Incidence of EAD or DAD was analyzed using Fisher's exact test. *P < 0.05, **P < 0.01.

We then examined whether SPX still increases late *I*_CaL_ and APDs in cardiac myocytes from adult mouse (6 month old), which is lack of native *I*_Kr_. We performed experiments similar to that shown in Fig 2. Fig 8A and 8C show that late *I*_CaL_ in adult mouse ventricular myocytes was significantly increased by exposure to 300 μM SPX for 10 min. Interestingly, SPX-induced reduction of peak *I*_CaL_ in adult mouse ventricular myocytes was not so pronounced as that observed in neonatal cardiac myocytes (Fig 8B). Consistent with increase in late *I*_CaL_, AP prolongation was observed when adult mouse cardiac myocytes were treated with SPX (300 μM) (Fig 8D & 8E). Fig 8E summarizes these results and shows that SPX prolonged APD_90_ over a range of stimulation rate in adult mouse cardiomyocytes. In addition, with exposure to SPX, triggered beats arising from early and delayed afterdepolarizations were observed in all cardiac myocytes examined at slow stimulation rate (n = 4); an example is shown in Fig 8F. In the absence of drug exposure, no afterdepolarizations were observed in cells (n = 4, Fig 8G). These findings exclude the potential E4031 effect and support the idea that SPX can increase late *I*_CaL_ and APDs in cardiac myocytes. Recently it was demonstrated that chronic exposure to some *I*_Kr_ blockers also increases cardiac late Na^+^ current, which is probably regarded as another mechanism for the drug-induced Q-T prolongation and TdP in patients chronically exposed to (non)-cardiac drugs in clinics [19]. We examined whether chronic exposure (5 hrs) to SPX enhances late Na^+^ currents in adult mouse ventricular myocytes. However no differences in late Na^+^ currents were observed between control and SPX-treated cells (S3 Fig), suggesting that the SPX effect on APs could not be attributed to a change in late Na^+^ currents.

## Discussion

It has been argued that the extent of *I*_Kr_ block is imperfect at best as a predictor of effects of a drug in a human subject [20, 21]. Proposed reasons for this discrepancy include a time-dependent effect on biosynthesis of hERG channel or on cell surface trafficking [22, 23] or failure of in vitro testing to consider other ion channel actions such as *I*_CaL_ or late Na^+^ currents [19, 24]. Our data showed that SPX markedly prolonged APD in a concentration-dependent manner in cardiac myocytes that lack *I*_Kr_, suggesting that SPX can be arrhthmogenic via an *I*_Kr_-independent pathway. SPX reduced peak *I*_CaL_ but augmented late *I*_CaL_ recorded several hundred milliseconds after a step depolarization and thus associated with APD prolongation. This effect is not seen with *I*_Kr_ blockers not associated with severe arrhythmias (ciprofloxacin, enoxacin, ofloxacin, and levofloxacin). We further showed that an antifungal agent ketoconazole, a potent *I*_Kr_ blocker without severe arrhythmias reduced both peak and late *I*_CaL_, suggesting a close relationship between late *I*_CaL_ and arrhythmogenesity. Detailed analysis showed that SPX treatment reduced the Ca^2+^-dependent component of steady-state inactivation, indicating that SPX attenuated CDI. Consequently, the steady-state levels of *I*_CaL_ were increased in the presence of SPX compared to that of the control. Consistent with the observed SPX-induced CDI attenuation, SPX had little effect on the inactivation time constant and steady-state inactivation once extracellular Ca^2+^ was replaced with Ba^2+^, a scenario in which essentially all inactivation is voltage dependent. The progressive use-dependent decrease of *I*_CaL_, which was assessed by applying repetitive voltage pulses at 2Hz, was less pronounced in the presence of SPX, indicating the positive effect of SPX on *I*_CaL_ occurred in a use-dependent manner. The recovery from inactivation of *I*_CaL_ was not altered by SPX. Taken together, our data suggest that SPX attenuates CDI, and the resulting slower *I*_CaL_ decay might contribute to SPX‐associated EAD and TdP.

The positive effects of SPX on *I*_CaL_ were concentration dependent, and SPX started to slow Ca^2+^ channel inactivation at a treatment concentration of 10 μM (Fig 3). Moreover, SPX-induced APD prolongation in the presence of E4031 was evident at 30 μM (Fig 1). Because the steady-state plasma concentration of SPX in healthy volunteers and patients was 1.8 μM and the hERG IC_50_ value is 18 μM [16], these results suggest that the slowing of *I*_CaL_ inactivation may attributable to SPX-induced LQT or arrhythmia under clinical conditions.

We demonstrated that SPX attenuated late *I*_CaL_ inactivation, especially at depolarized potentials (≥0 mV) without voltage shift of steady-state curve. Therefore, inactivation curve was more U-shaped in the presence of SPX (Fig 4). These results suggest that SPX specifically interrupts the Ca^2+^-dependent component of *I*_CaL_ inactivation, having little effect on the voltage-dependent component. In support of this hypothesis, when Ba^2+^ was used as the *I*_CaL_ charge carrier (Fig 5), SPX-induced inactivation slowing and the consequent increase in the late *I*_CaL_ were abolished (Fig 5).

Our data showed that SPX treatment reduced peak *I*_CaL_ amplitude as well as slowed its inactivation_._ These two changes have opposing effects on APD. However, the results of previous studies suggest that the kinetics of *I*_CaL_ inactivation, rather than the amplitude modulates its effects on the APD restitution slope and reentry [25]. Consistent with this concept, SPX induced APD prolongation, because of the dominance of suppressed *I*_CaL_ inactivation in controlling APD compared to the ongoing reduction of *I*_CaL_ amplitude. In a context in which there is a concomitant reduction of repolarizing current, which should shorten APs, the slowed *I*_CaL_ decay is an important factor in tipping the balance towards EAD formation.

Although the precise molecular sites that are responsible for the CDI is not entirely clear yet, it has been demonstrated that two kinds of Ca^2+^-binding sites model (i.e., high-affinity slow and low-affinity fast kinetic binding sites) successfully simulated the CDI obtained by experiments [26, 27]. The high affinity binding site was expected to be present very near at inner channel mouth and not to be accessible by intracellular Ca^2+^ buffers such as EGTA or BPATA. Therefore, this CDI is attributable by the influx of Ca^2+^ itself and can’t be excluded by pipette EGTA or BAPTA. It can only be excluded by the substitution of Ca^2+^ with other ions such as Ba^2+^ for the charge carrier. Classically, the ‘domain’ model of CDI can explain well this high affinity Ca^2+^ binding site model [26–28]. The low-affinity Ca^2+^ binding site can explain well the ‘shell’ model of CDI, in which global increase in cytosolic [Ca^2+^]i mediates the CDI [27, 28]. The Ca^2+^ that are released from intracellular store such as sarcoplasmic reticulum (SR) took the biggest part in the ‘shell’ or ‘low-affinity Ca^2+^ binding site’ models [27]. Therefore, the release-dependent inactivation (RDI) was primarily responsible for CDI in the ‘low-affinity Ca^2+^ binding site’ model [27]. High concentrations of pipette Ca^2+^ buffer such as BAPTA can effectively exclude this CDI that is mediated by the low-affinity Ca^2+^ binding site. Since we used pipette solution with 10 mM EGTA in the present study, it is expected that SR Ca^2+^ is depleted and the CICR is largely prevented. Therefore, the CDI of this study is thought to be primarily mediated by the high affinity Ca^2+^ binding site probably very near at the channel mouth. Taken together, the slowing of inactivation time course of *I*_CaL_ by SPX was not secondary phenomenon due to the decreases in peak *I*_CaL_ and intracellular [Ca^2+^], but due to SPX-induced specific inhibition of CDI that is mediated by high affinity Ca^2+^-binding site (that is, an EGTA-insensitive site). Moreover, lack of effects on the inactivation time courses by ketoconazole (Fig 2), at concentrations that inhibit the peak *I*_CaL_ similarly to those of SPX, also indicates that the CDI of the present study is not mediated by the global intracellular [Ca^2+^] increase.

In conclusion, the present findings demonstrate the role of *I*_CaL_ in SPX-induced APD prolongation. Our results suggest that modification of *I*_CaL_ properties, in addition to *I*_Kr_ antagonistic activities, should be considered when assessing the proarrhythmic potential of drugs. Especially new drug evaluation will need to look beyond effect on peak *I*_CaL_ and examine drug effects on late *I*_CaL_ of which perturbation induces abnormal repolarization.

## Supporting Information

S1 Fig **L-type and T-type Ca^2+^ channels in neonatal rat cardiomyocytes** A, Ca^2+^ currents were elicited by depolarizing voltage steps from a holding potential of −80 mV in the absence and presence of nifedipine (1 μM) or nifedipine (1 μM) plus NiCl_2_ (100 μM). B, Current–voltage (*I*–V) relationships of the peak Ca^2+^ current (holding potential −80 mV) in the absence and presence of Ca^2+^ channel inhibitors (black, control; red, Nifedipine; green, Nifedipine + NiCl_2_. C, Ca^2+^ currents were elicited by depolarizing voltage steps from a holding potential of −50 mV in the absence and presence of nifedipine (1 μM). D, Current–voltage (*I*–V) relationships of the peak Ca^2+^ currents in the absence and presence of nifedipine (holding potential −50 mV; black, control; red, nifedipine).
(TIF)

S2 Fig **Effects of quinolones on *I*_CaL_** Representative traces showing the effect of 1 mM ciprofloxacin (A), 1 mM enoxacin (B), 1 mM ofloxacin (C), and 1 mM levofloxacin (D) on *I*_CaL_. Representative traces of *I*_CaL_ during a 200-ms voltage-clamp pulse from −40 to 0 mV before (black) and after (red) exposure to 300 μM SPX. Lower panels (E−H) summarize the concentration-response of the quinolones.
(TIF)

S3 Fig **Chronic exposure to SPX does not increase late Na^+^ current** A, Examples of Na^+^ current recorded 5 hours after isolation in the absence (vehicle; left), or in the presence of SPX (right). The selective late current blocker ranolazine did not affect Na^+^ current in SPX-treated cells as well as cells under control condition. B, Summary data show that there was no effect on late Na^+^ current of 5-hour exposure to SPX in adult mouse ventricular myocytes.
(TIF)

## Abbreviations

AP: action potential
APD: action potential duration
CDI: Ca^2+^-dependent inactivation
EAD: early afterdepolarization
hERG: human *Ether*-*à*-*go*-*go*-related gene
*I*_CaL_: L-type Ca^2+^ current
*I*_CaT_: T-type Ca^2+^ current
*I*_Kr_: rapidly activating delayed-rectifier K^+^ current
LQT: long QT
SPX: sparfloxacin
TdP: Torsade de Pointes
VT: ventricular tachycardia
VDI: voltage-dependent inactivation
