# Supplementary figures and images for: Impaired Inactivation of L-Type Ca2+ Current as a Potential Mechanism for Variable Arrhythmogenic Liability of HERG K+ Channel Blocking Drugs
Source: PLoS One. 2016 Mar 1;11(3):e0149198. doi: 10.1371/journal.pone.0149198 (PMC4772914; doi:10.1371/journal.pone.0149198)

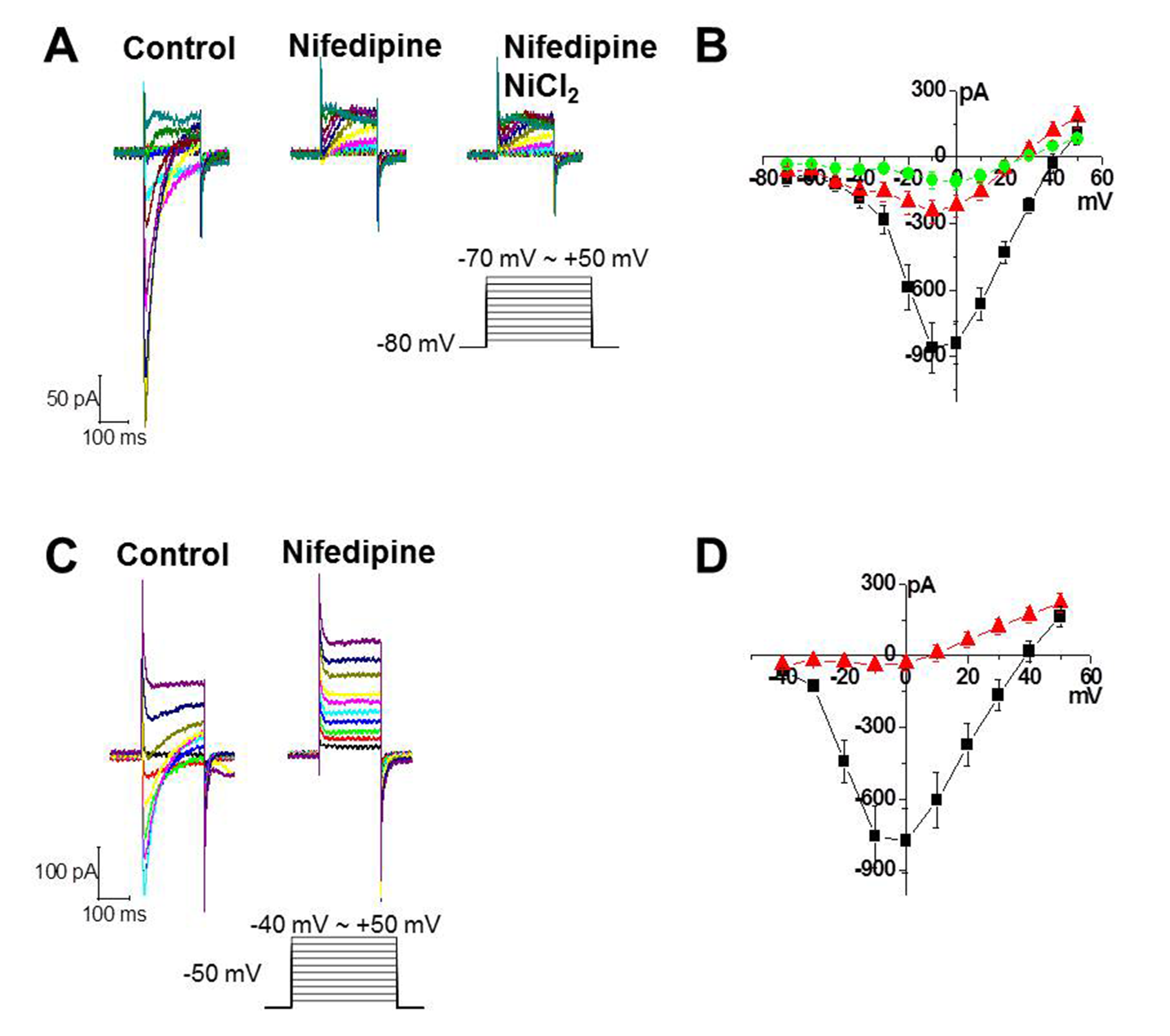

Supplement: S1 Fig — L-type and T-type Ca2+ channels in neonatal rat cardiomyocytes A, Ca2+ currents were elicited by depolarizing voltage steps from a holding potential of −80 mV in the absence and presence of nifedipine (1 μM) or nifedipine (1 μM) plus NiCl2 (100 μM). B, Current–voltage (I–V) relationships of the peak Ca2+ current (holding potential −80 mV) in the absence and presence of Ca2+ channel inhibitors (black, control; red, Nifedipine; green, Nifedipine + NiCl2. C, Ca2+ currents were elicited by depolarizing voltage steps from a holding potential of −50 mV in the absence and presence of nifedipine (1 μM). D, Current–voltage (I–V) relationships of the peak Ca2+ currents in the absence and presence of nifedipine (holding potential −50 mV; black, control; red, nifedipine). (TIF) [file pone.0149198.s001.tif]

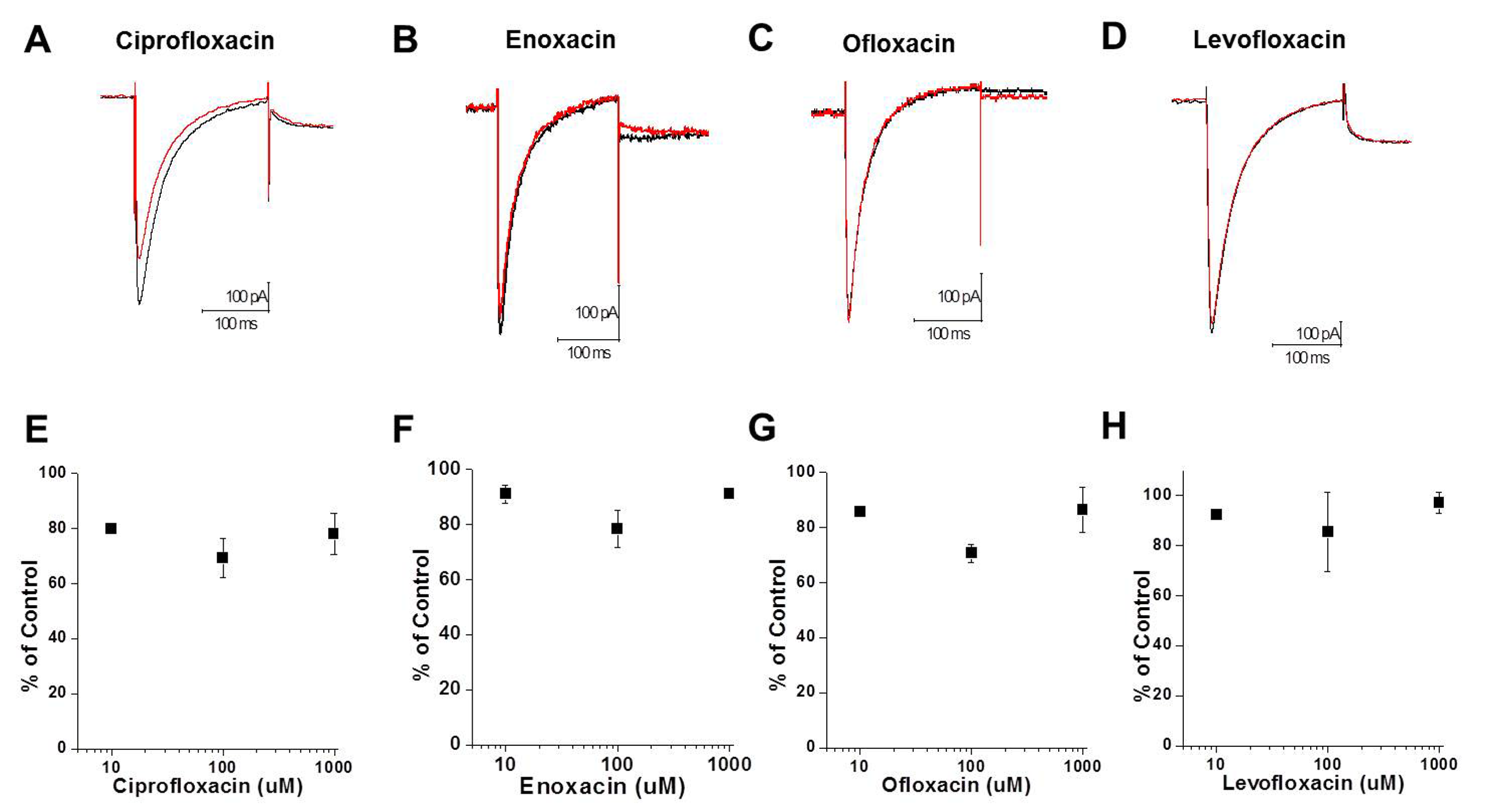

Supplement: S2 Fig — Effects of quinolones on ICaL Representative traces showing the effect of 1 mM ciprofloxacin (A), 1 mM enoxacin (B), 1 mM ofloxacin (C), and 1 mM levofloxacin (D) on ICaL. Representative traces of ICaL during a 200-ms voltage-clamp pulse from −40 to 0 mV before (black) and after (red) exposure to 300 μM SPX. Lower panels (E−H) summarize the concentration-response of the quinolones. (TIF) [file pone.0149198.s002.tif]

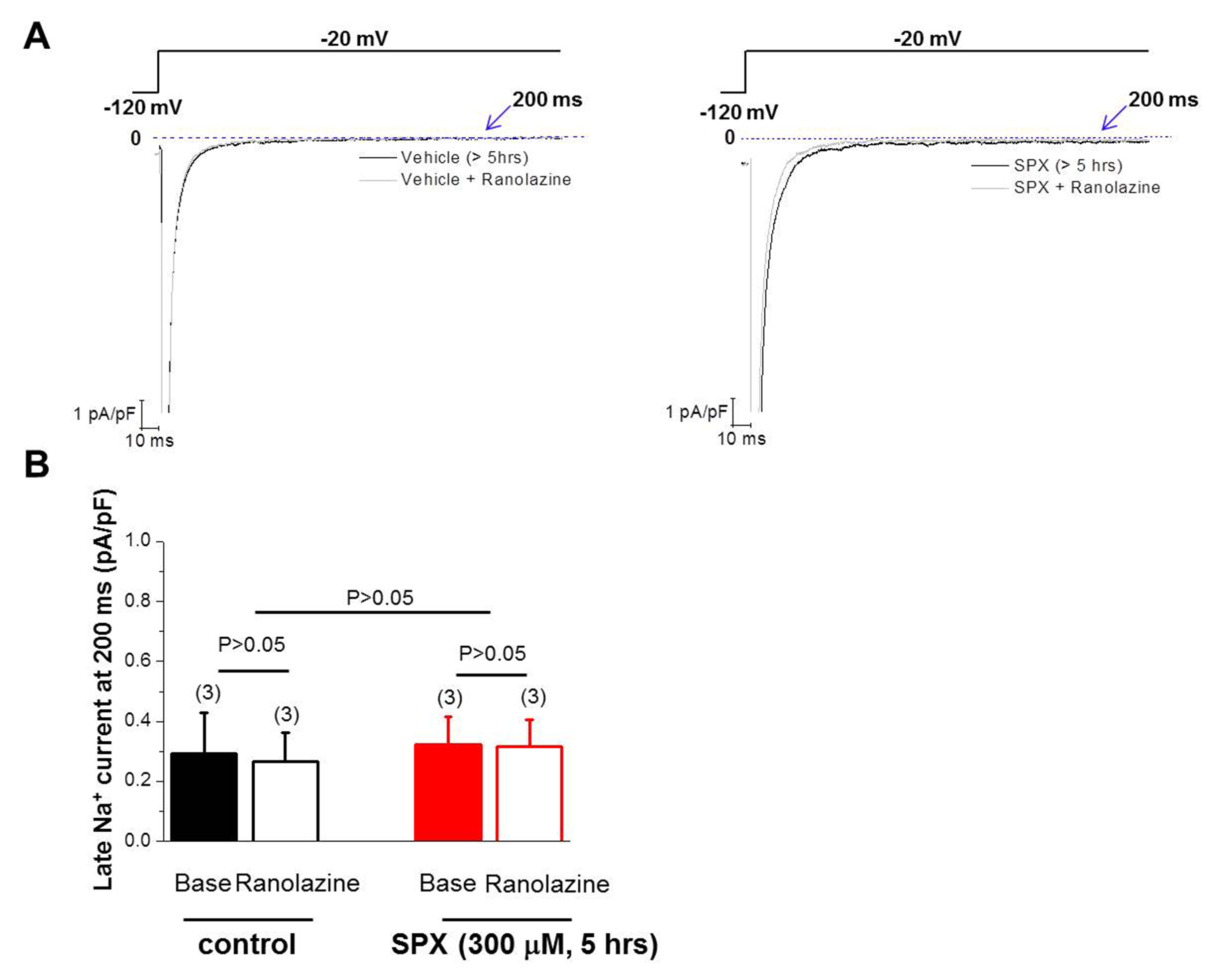

Supplement: S3 Fig — Chronic exposure to SPX does not increase late Na+ current A, Examples of Na+ current recorded 5 hours after isolation in the absence (vehicle; left), or in the presence of SPX (right). The selective late current blocker ranolazine did not affect Na+ current in SPX-treated cells as well as cells under control condition. B, Summary data show that there was no effect on late Na+ current of 5-hour exposure to SPX in adult mouse ventricular myocytes. (TIF) [file pone.0149198.s003.tif]
